# Siblings with megalencephalic leukoencephalopathy with subcortical cysts van der Knaap disease

**DOI:** 10.1259/bjrcr.20200150

**Published:** 2021-06-19

**Authors:** Saanida M P, Lin Varghese, Rinu Susan Thomas, Sandeep Govindan Prasad

**Affiliations:** 1 Department of Radiology, Government Medical College, Kozhikode, Kerala, India

## Abstract

Cerebral leukoencephalopathy and megalencephaly with subcortical cysts (also known as van der Knaap disease) is an autosomal recessive condition. The disease was initially described in India and Netherlands independently and seems to have highest incidence in Indian Agrawal community and Turkish population.^1^ The objective of this study is to document the case of two siblings with this condition, from a non-Agrawal Indian community and briefly describe the imaging features of this condition. Two siblings, born out of a third-degree consanguineous marriage, with simple focal seizures were subjected to MRI with diffusion-weighted imaging and spectrometry. The findings were compared to diseases with similar clinical presentation. Subcortical cysts initially involving anterior temporal lobes and subsequently frontal and parietal lobes, sparing of central white mater, small N acetyl aspartate peak and diffusion facilitation were the imaging findings. The imaging findings were consistent with the diagnosis of the rare genetic disorder- Cerebral leukoencephalopathy and megalencephaly with subcortical cysts.

## Background

Autosomal recessive cerebral leukoencephalopathy and megalencephaly with subcortical cysts, also eponymously known as van der Knaap syndrome is a rare genetic disorder of infantile onset. First described by Dr van der Knaap in 1995,^2^ this disease was found to have highest incidence in Agrawal community in northern India and Turkish population.^1^ It is a neurodegenerative disorder characterized by infantile onset megalencephaly and cerebral leucoencephalopathy. Despite extensive changes in imaging, the neurological symptoms are mild and the course of functional deterioration is extremely slow.^2^ The degree of macrocephaly is variable and can be 4–6 SD above the mean. Almost all patients have epilepsy from an early age.^3^ Some patients die in their second or third decades but few may live till fourth decade. This is in contrast to other neurological disorders with onset in infancy, like Canavan disease and Alexander disease, where neurological dysfunction is severe, there is rapid clinical deterioration and death occurs within the first few years of life. It is reported that MRI findings are characteristic for marked reduction of N-acetyl aspartate, creatine, and choline with normal values for myoinositol, consistent with axonal loss and astrocytic proliferation in MR spectroscopy.^4^ We report two cases of van der Knaap syndrome in siblings from a non-Agrawal community in southern Indian state of Kerala.

## Case report

**Figure 1.**
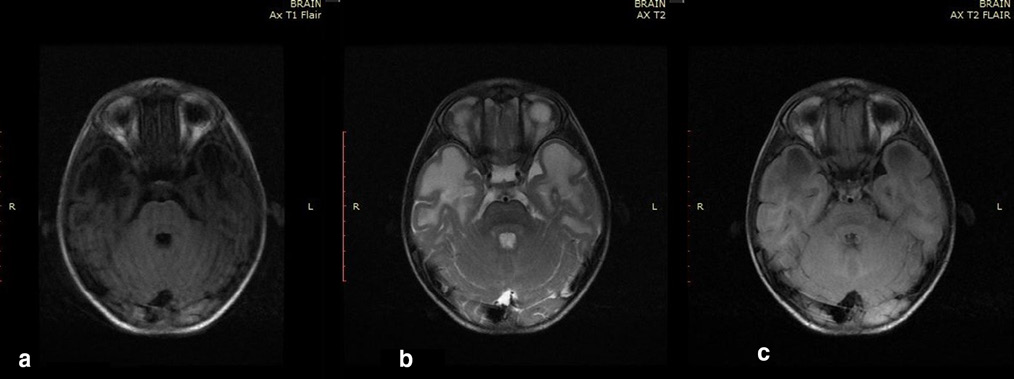
Axial *T*_1_WI image of brain of the first child showing bilateral subcortical cysts appearing hypo intense similar to CSF intensity affecting anterior temporal lobes (Figure 1a), axial *T*_2_WI image of brain showing bilateral subcortical cysts appearing hyperintense similar to CSF intensity affecting anterior temporal lobes (Figure 1b) with suppression on axial FLAIR images (Figure 1c). CSF, cerebrospinal fluid; FLAIR, fluid attenuated inversion recovery.

**Figure 2.**
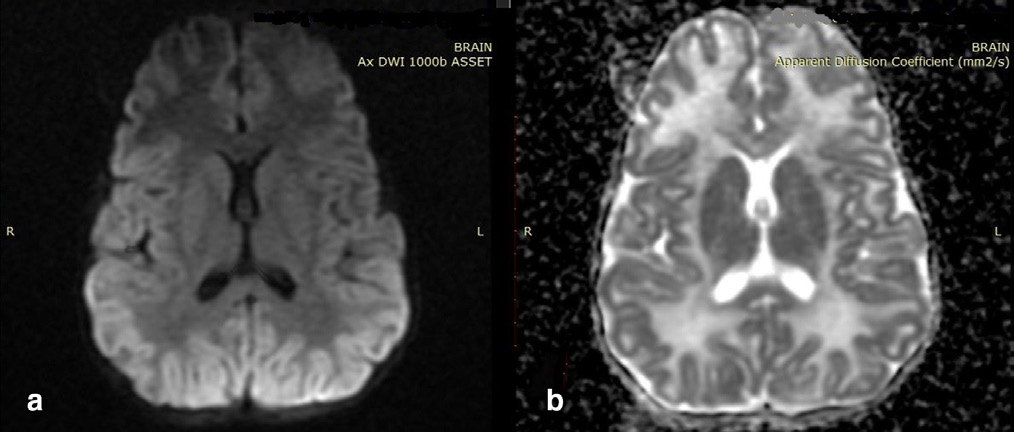
Axial diffusion-weighted image of the first child showing hypointensity involving bilateral anterior temporal lobes (Figure 2a) and corresponding areas in apparent diffusion coefficient image appearing bright (Figure 2b).

**Figure 3.**
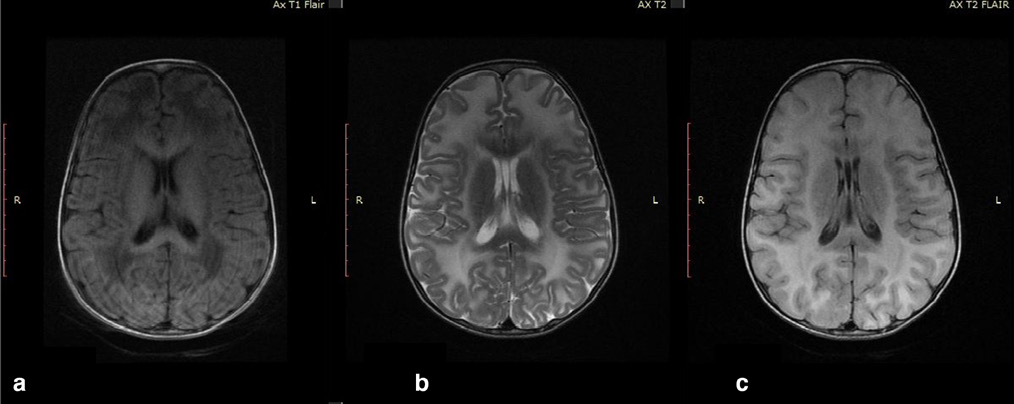
Figure 3a - axial *T*_1_WI image of brain of the first child showing involvement of subcortical U fibers bilaterally. Figure 3b - axial *T*_2_WI image of brain showing involvement of subcortical U fibers bilaterally with suppression in FLAIR (Figure 3c) FLAIR, fluid attenuated inversion recovery.

**Figure 4.**
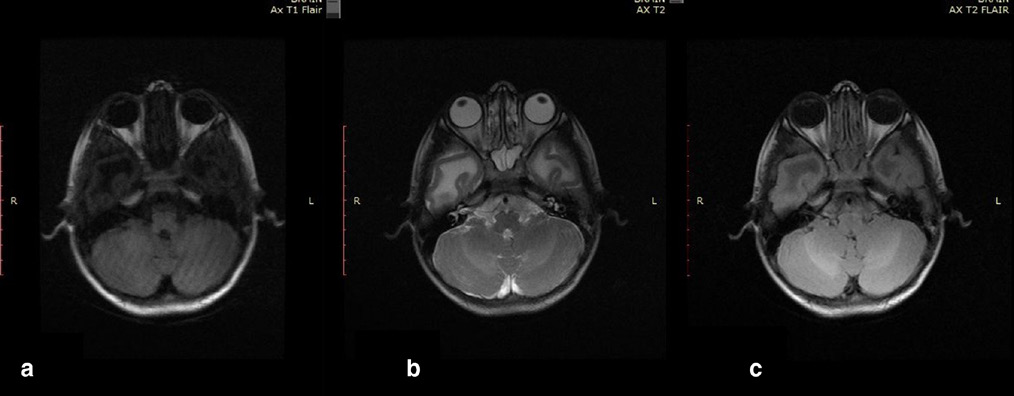
Axial *T*_1_WI (Figure 4a), Axial *T*_2_WI (Figure 4b) and axial FLAIR image (Figure 4c) of brain of the first child showing sparing of cerebellar and deep white matter. FLAIR, fluid attenuated inversion recovery.

**Figure 5.**
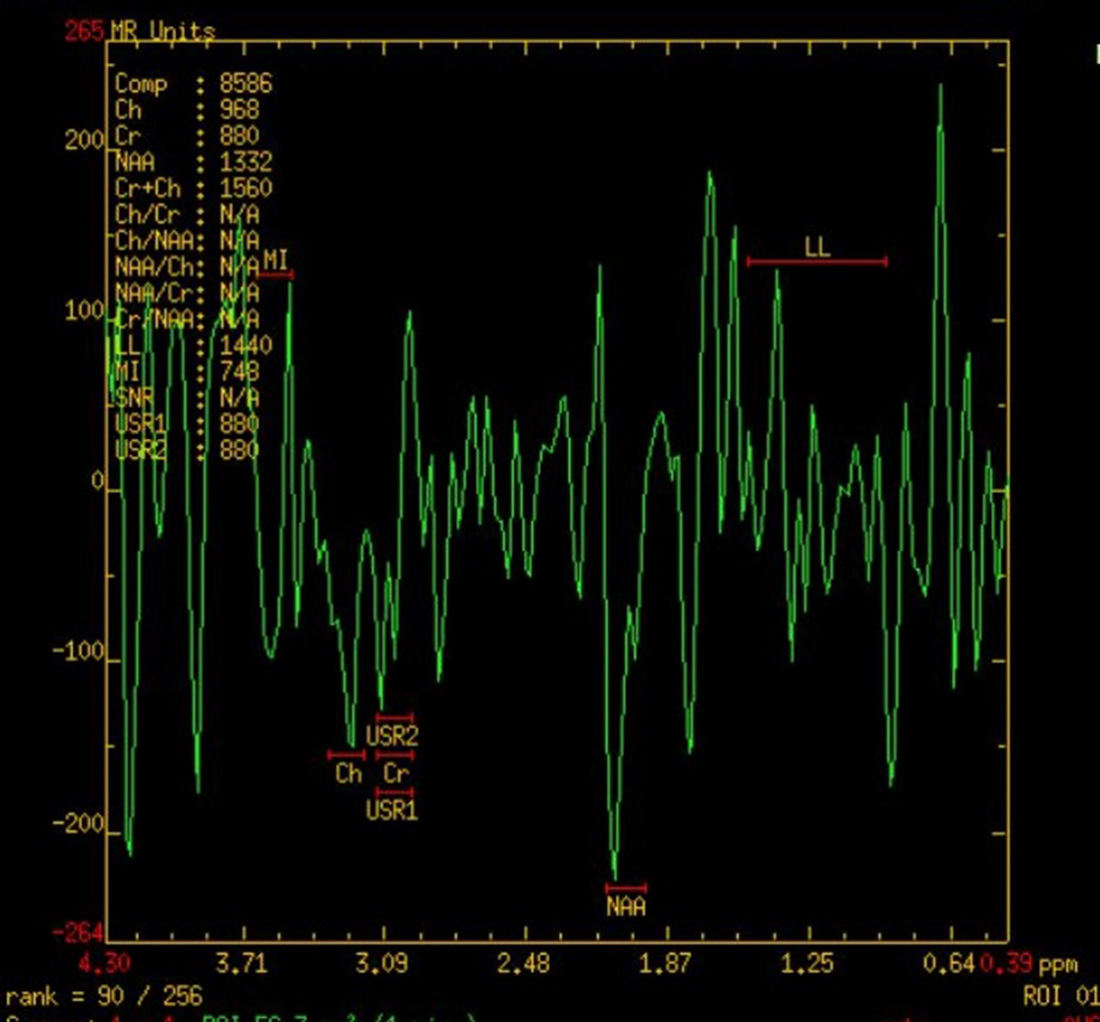
MR spectroscopic image of brain of the first child showing NAA dip in affected white matter. NAA, N acetyl aspartate.

Case 1: 4-year-old male child with a history of recurrent falls for 2 years which started after an episode of left-sided simple focal seizure. Two episodes of left-sided simple focal seizures have occurred since. The antenatal period was uneventful. Post-natally, the child underwent phototherapy for raised serum bilirubin. The gross motor development was delayed with head control attained at 5 months of age, stood with support at 18 months, and walked without support at 2 years. Transferring objects appeared at 7 months, pincer grasp at 1 year, and ability to speak small sentences appeared at the age of 1.5 years. Upon presentation, he was able to climb stairs, one at a time and to copy a circle. Bowel and bladder control is normal. The parents of the child had third-degree consanguineous marriage. Consanguinity is extremely uncommon in their community and similar illness has not been reported in their family. A similar history of delayed developmental milestones was present in the elder sibling. On examination, the child was alert, active and afebrile. The head appeared enlarged. Head circumference was 51.5 cm (between 85th and 97th centile). Height was 94.5 cm and weight was 10 kg (less than 1st centile). Mid-arm circumference was normal for age. On neurological examination, he had decreased tone of muscles and wide based, swaying gait. All the routine blood investigations were normal. Electroencephalogram (EEG) revealed epileptiform discharges from right central region. MRI showed megalencephaly, bilateral subcortical cysts of cerebrospinal fluid (CSF) intensity affecting the anterior temporal lobes (Figure 1), diffusion facilitation in bilateral anterior temporal lobes (Figure 2), involvement of subcortical U fibers(Figure 3) and relative sparing of deep and cerebellar white matter (Figure 4). MR spectroscopy revealed N acetyl aspartate (NAA) dips in affected white matter (Figure 5).

Case 2: 6-year-old female sibling of patient described earlier, had an episode of left sided simple focal seizure 4 years back. The antenatal and post-natal period of the child was uneventful. The gross motor development was delayed with head control attained at 5 months of age, standing with support attained at 1 years 3 months, and walking without support at 2 years. At presentation, she was able to climb stairs one at a time and copy a circle. Bladder control and vision were normal. On examination, the child was alert, active and afebrile. The head appeared enlarged. Head circumference was 51.5 cm (between 97th and 50th centile). Height was 94.5 cm and weight was 10 kg (less than 1st centile). Mid-arm circumference was normal for age. On neurological examination, she had decreased tone of muscles and wide based swaying gait. All the routine blood investigations were normal. EEG revealed epileptiform discharges from right central region.

**Figure 6.**
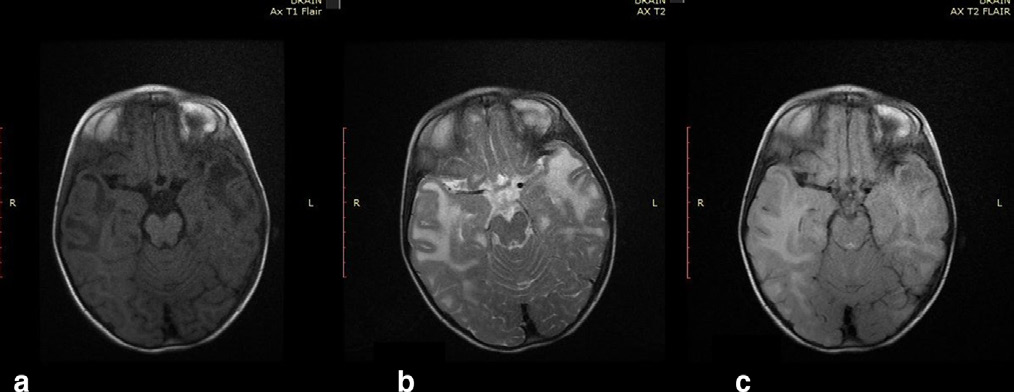
Axial *T*_1_WI image of brain of the second child showing bilateral sub cortical cysts appearing hypo intense similar to CSF intensity affecting anterior temporal lobes (Figure 6a), axial *T*_2_WI image of brain showing bilateral sub cortical cysts appearing hyper intense similar to CSF intensity affecting anterior temporal lobes (Figure 6b) with suppression on axial FLAIR images (Figure 6c) CSF, cerebrospinal fluid; FLAIR, fluid attenuated inversion recovery.

MRI showed megalencephaly, bilateral subcortical cysts of CSF intensity affecting the anterior temporal lobes (Figure 6), diffusion facilitation in bilateral anterior temporal lobes, involvement of subcortical U fibers and relative sparing of deep and cerebellar white matter (Figure 6). MR spectroscopy revealed NAA dips in affected white matter.

**Figure 7.**
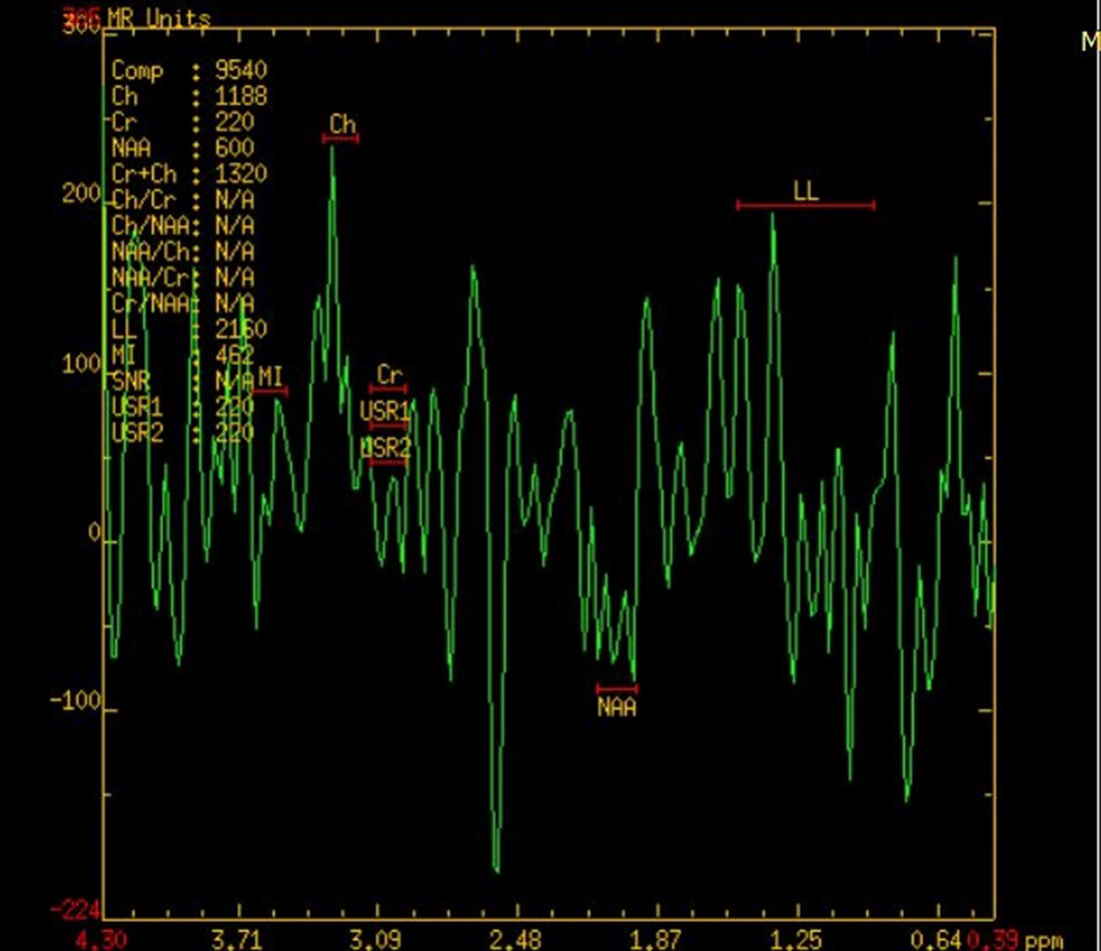
MR spectroscopic image of brain of the second child showing NAA dip in affected white matter. NAA, N acetyl aspartate.

## Discussion

Megalencephalic leukoencephalopathy with subcortical cysts, also eponymously known as van der Knaap syndrome, is an autosomal recessive disorder with infantile onset. Despite a very abnormal imaging study, the disease is clinically mild in presentation. Patients usually presents in first year of life with megalencephaly, but a presentation in second year of life with mild delay in developmental milestones is not uncommon. Even though the head circumference stabilizes over time, there is a gradual deterioration with increasing spasticity, dysarthria, ataxia and dementia. This leads to most patients being bound to wheel chair by their first decade of life, which is also associated with a slow cognitive deterioration.

Once the condition is suspected clinically, MRI features are diagnostic of the condition. Imaging reveals near complete absence of myelin in subcortical white matter. Central white matter sparing is also seen and corpus callosum, brainstem, internal capsule and occipital lobes are particularly spared. Swelling of subcortical white matter with enlargement of gyri is seen. Subcortical cysts that initially develop in anterior temporal lobes and subsequently in frontal and parietal regions is a characteristic finding.^3^ Even though cerebellum is relatively spared, mild cerebellar atrophy and some degree of T2 prolongation is present in most patients. Diffusion studies show diffusion facilitation. MR spectroscopy reveals particularly low NAA levels.

Two phenotypes are described, namely classic phenotype (MLC 1 or MLC 2A) associated with biallelic pathogenic variants in MLC 1 and HEPACAM and improving phenotype (MLC 2B) associated with heterozygous HEPACAM pathogenic variant.^5^ In contrast to classic phenotype, improving phenotype megalencephalic leukoencephalopathy may show improvement in motor symptoms after 1 year and striking improvements in brain MRI over time. Cerebellar white matter in the classic phenotype shows signal alterations, although not swollen while improving phenotype of the disease shows no signal changes in the cerebellum. Mutations of the MLC 1 gene at 22qtel is seen in 4 out of 5 of patients, whereas 20% of patients are devoid of this mutation. The diagnosis of megalencephalic leukoencephalopathy can be made on the basis of typical clinical and MRI features.^5^ Identification of biallelic pathogenic variant of MLC1 or HEPACAM and heterozygous pathogenic variant of HEPACAM can confirm the diagnosis of classic phenotype and improving phenotype of the disease respectively when clinical features and imaging findings are inconclusive.^5^ Once diagnosis has been established with typical clinical and MRI features, genetic testing can be used for family studies.^5^

**Table 1.** Summary of cases

		Case 1	Case 2
Age and gender		**4 years, Male**	**6 years, Female**
Initial motor development		**Mildly delayed**	**Mildly delayed**
Initial mental development		**Mildly delayed**	**Mildly delayed**
Present motor function		**Mildly delayed**	**Mildly delayed**
Present intellectual function		**Mildly delayed**	**Mildly delayed**
Epilepsy (first seizure)		**2 years of age**	**2 years of age**
Consanguinity of parents		**Present**	**Present**
Family history		**Absent**	**Absent**
MRI Findings	Supratentorial white matter (WM) involvement	**Diffuse**	**Diffuse**
	WM swelling	**Present**	Present
	Sparing of periventricular WM	Occasional	Occasional
	Sparing of subcortical WM	Occasional	Occasional
	T2 hyperintense & FLAIR suppressed cystic areas	Anterior Temporal, *R* > L	Anterior Temporal, *R* = L
	Cerebellar WM involvement	**Relative sparing**	Relative sparing
	Involvement of gray matter structures	**Not Involved**	Not Involved
	NAA dip in MR spectroscopy	**Present**	**Present**

FLAIR, fluid attenuated inversion recovery; NAA, N acetyl aspartate.

The differential diagnoses of this condition are not extensive. They are Canavan disease, Alexander disease, Vanishing white matter disease and Pelizaeus-Merzbacher disease. The clinical features and course of these disorders are usually different from those of Megalencephalic leukoencephalopathy with subcortical cysts. If the head circumference is well within the normal limits at age 1 year, it is highly unlikely that the infant has this disease and the more likely possibility would be leukoencephalopathy with subcortical cysts^6^ None of these disorders share all the MRI characteristics of megalocephalic leukoencephalopathy with subcortical cysts. (Table 2).

**Table 2.** Comparison with similar diseases^3^

	CANAVAN DISEASE	ALEXANDER DISEASE	VANISHING WHITE MATTER DISEASE	PELIZAEUS-MERZBACHER DISEASE	MEGALENCEPHALIC LEUKOENCEPHALOPATHY WITH SUBCORTICAL CYSTS
Age of onset	First year	First year	2–6 years	First year	Second year
Predominant presentation	Hypotonia, macrocephaly, seizures, delayed psychomotor development, spasticity, optic atrophy	Macrocephaly, developmental delay, progression to psychomotor retardation	Progressive ataxia and spastic diplegia with relapsing-remitting phases, development of bulbar symptoms, optic atrophy, and epilepsy	Nystagmus, Hypotonia, extrapyramidal hyperkinesia, spasticity	Macrocephaly, delay in attaining developmental milestones. spasticity, dysarthria, ataxia, and dementia
Progression	Rapid (Death by age of 2 years)	Rapid and lethal	Slow	Variable	Slow
Inheritance	AR^a^	AD^b^	AR^a^	XR^c^	AR^a^
Imaging	Involvement of globus pallidus and thalamus, no subcortical cysts. MRS- large NAA peak, diffusion restriction +	T1 and T2 prolongation in the medulla, pons, and middle cerebellar peduncles, intense post contrast enhancement, cavitation /cysts, diffusion facilitation, small NAA peak	Diffuse white matter involvement with cystic changes, immediate subcortical and temporal sparing, Cerebellar white matter involvement, No contrast enhancement.	T2 lengthening throughout the brain, cerebellum markedly atrophic	Subcortical cysts, sparing of central white matter. MRS- small NAA peak, diffusion facilitation

MRS, MR spectroscopy; NAA, N acetyl aspartate.

Foot note a– autosomal recessive

b – autosomal dominant

c – X – linked recessive

This article describes a rare genetic neurological disorder being reported in two siblings from a non-Agrawal community in India. The main limitation of this study is that MLC gene study could not be done.

## Conclusions

Megalencephalic leukoencephalopathy with subcortical cysts also known as van der Knaap disease is commonly reported from Turkish population and Agrawal community in India where consanguineous marriages occur frequently. However, it may also be reported in children from other communities when parents have some degree of consanguinity.

Despite initially mild presentation in first year of life, disease is noted to have a slow progressive nature which severely impairs quality of life over time. The diagnosis of megalencephalic leukoencephalopathy is made on the basis of clinical and MRI features. Awareness of the entity, its imaging features and inheritance pattern would aid in conducting genetic studies and counseling.

## Learning points

1. This article describes a rare genetic neurologic disorder in two siblings from a non-Agrawal community in India where an epidemiologic predisposition has not been described.
2. MRI features including near total absence of myelin in subcortical white matter with central sparing and subcortical cysts initially involving anterior temporal lobes enable the radiologist to make a fairly specific diagnosis when used in conjunction with clinical features.
3. This study would prompt conducting genetic studies and counseling to prevent further occurrence of the disease.
